# Impact of facial and truncal acne on quality of life: A multi-country population-based survey

**DOI:** 10.1016/j.jdin.2021.03.002

**Published:** 2021-04-27

**Authors:** Jerry Tan, Stefan Beissert, Fran Cook-Bolden, Rajeev Chavda, Julie Harper, Adelaide Hebert, Edward Lain, Alison Layton, Marco Rocha, Jonathan Weiss, Brigitte Dréno

**Affiliations:** a Schulich School of Medicine and Dentistry, Western University Canada, Windsor, Ontario, Canada; b Department of Dermatology, University Hospital Carl Gustav Carus, TU Dresden, Dresden, Germany; c Mount Sinai Department of Dermatology, New York, New York; d Galderma, La Tour-de-Peilz, Switzerland; e The Dermatology and Skin Care Center of Birmingham, Birmingham, Alabama; f The University of Texas Medical School – Houston, Houston, Texas; g Austin Institute for Clinical Research, Pflugerville, Texas; h Hull York Medical School, York University, Heslington, York, United Kingdom; i Federal University of São Paulo, São Paulo, Brazil; j Georgia Dermatology Partners (Formerly, Gwinnett Dermatology, PC), Snellville, Georgia; k Unité Thérapie Cellulaire et Génique, Faculté de Médecine de Nantes, CHU Nantes - Place Alexis Ricordeau, Nantes, France

**Keywords:** CompAQ, dermatology life quality index (DLQI), facial acne, patient-reported outcomes, quality of life, truncal acne, CDLQI, children's dermatology life quality index, CI, confidence interval, CompAQ, Comprehensive Acne Quality of Life, DLQI, dermatology life quality index, F, facial acne only, F+T, combined facial and truncal acne, HRQoL, health-related quality of life, OR, odds ratio

## Abstract

**Background:**

Acne confers an increased risk of physical, psychiatric, and psychosocial sequelae, potentially affecting multiple dimensions of health-related quality of life (HRQoL). Morbidity associated with truncal acne is poorly understood.

**Objective:**

To determine how severity and location of acne lesions impact the HRQoL of those who suffer from it.

**Methods:**

A total of 694 subjects with combined facial and truncal acne (F+T) and 615 with facial acne only (F) participated in an online, international survey. Participants self-graded the severity of their acne at different anatomical locations and completed the dermatology life quality index (DLQI).

**Results:**

The F+T participants were twice as likely to report “very large” to “extremely large” impact on HRQoL (ie, DLQI > 10 and children's DLQI [CDLQI] > 12) as compared with the F participants (DLQI: odds ratio [OR] 1.61 [95% confidence interval {CI} 1.02-2.54]; CDLQI: OR 1.86 [95% CI 1.10-3.14]). The impact of acne on HRQoL increased with increasing acne severity on the face (DLQI and CDLQI *P* values = .001 and .017, respectively), chest (*P* = .003; *P* = .008), and back (*P* = .001; *P* = .028).

**Limitations:**

Temporal evaluation of acne impact was not estimated.

**Conclusions:**

Facial and truncal acne was associated with a greater impact on HRQoL than facial acne alone. Increasing severity of truncal acne increases the adverse impact on HRQoL irrespective of the severity of facial acne.

**Capsule Summary**

• Facial and truncal acne has a significant impact on emotional well-being and everyday life activities.
• The additional impact of truncal acne on quality of life implies that early and effective treatment of truncal acne is important to limit disease-related psychosocial sequelae.

## Introduction

Acne is an inflammatory disease of pilosebaceous units with an estimated global prevalence (all ages) of 9.4%, ranking it among the top 10 most prevalent conditions worldwide.^1^^,^^2^ It primarily affects the face (99%) and less frequently the chest or back (ie, approximately half of the cases with facial acne).^3^^,^^4^ This inflammatory disorder typically develops during the teen years, affecting up to 100% of adolescents, and can continue into adulthood; some affected individuals can present with chronic unremitting disease.^5^^,^^6^ Overall, acne can inflict lifelong physical, psychiatric, and psychosocial sequelae, affecting multiple dimensions of the health-related quality of life (HRQoL).^7^, ^8^, ^9^, ^10^, ^11^, ^12^, ^13^, ^14^, ^15^, ^16^, ^17^, ^18^, ^19^, ^20^, ^21^, ^22^, ^23^ The psychosocial impacts of acne have been estimated to be similar to those of other chronic diseases such as asthma, epilepsy, diabetes, or arthritis.^9^ Although the impact of acne on HRQoL correlates with disease severity, patients with mild disease can also present with significant HRQoL impairment.^24^

Most prior HRQoL studies on acne have focused on facial acne.^25^^,^^26^ However, just as with facial acne, acne can affect the chest and back with varying severity, and the location of acne has been shown to differentially impact the patient's HRQoL experience.^27^, ^28^, ^29^ Evaluation of acne severity and impact beyond facial involvement can provide a means to develop a comprehensive patient management strategy. More recently, acne grading scales^30^ and acne specific HRQoL measures inclusive of both facial and truncal acne were developed to facilitate these assessments.^31^^,^^32^

In this cross-sectional survey, the goal was to determine the extent to which acne location affects HRQoL. This study investigated whether HRQoL impairment differs between those with facial acne only (F) versus those with facial and truncal acne (F+T).

## Materials and methods

This was a cross-sectional, web-based survey of an online respondent panel aged ≥18 years (ie, Kantar LightSpeed GMI, Dynata, Toluna, M3, Lucid, BA) who had previously agreed to respond to health surveys about their medical condition(s) or those of their child. All participants of the study aged 13 to <18 years old were assented and permitted by their legal guardian. The research complied with General Data Protection Regulation, all international/local data protection legislation, and Insights Association/European Society for Opinion and Marketing Research/European Pharmaceutical Market Research Association/British Healthcare Business Intelligence Association. All subjects provided informed consent prior to participation. Minors were required to answer the survey questions themselves. The survey was administered in the native language of each country (ie, United States of America, Canada, France, Germany, Italy, and Brazil) between November 2019 and January 2020. Based on the formula [click through/panelists who received an email with the study link], the response rate of the survey was approximately 5%.

A quota sampling method based on geographic location was used to ensure that the sample of respondents was representative of acne populations in these countries. A weighting adjustment was applied at the country level if deviations were observed between the sample and the expected age and sex distribution of the acne population in these countries.^33^ Country weights were also used to account for population size. A comparison of key study results for weighted and unweighted data found no significant differences between both results’ analyses. This report is presented based on the weighted data.

After informed consent was obtained, the potential participants were asked to complete a sociodemographic questionnaire that was used to determine study eligibility. Inclusion criteria was defined as male or female subjects aged between 13 and 40 years who had self-reported a physician diagnosis of acne, who were currently being followed by a health care professional, and who were receiving prescription treatment for acne. The severity of the acne was assessed using a self-rated 6-category global acne grading system based on the Investigator Global Assessment for the face, which was modified to include the trunk (chest and back).^30^ To facilitate self-assessment of severity, photo-scales were provided as examples of severity for the face, chest, and back alongside text descriptions. Participants were required to have mild to very severe facial acne at the time of survey completion and moderate to very severe facial acne as their worst acne onset in the past 12 months to be included in the F group; to be included in the F+T group participants were also required to have the same level of severity on the chest and/or back at the time of survey completion and as their worst acne onset in the past 12 months.

The survey obtained information on demographics (eg, sex, age, and residential background) and clinical characteristics (eg, family history of acne, presence of acne signs/symptoms, the number of years living with the condition, body location and self-assessed severity of acne, current acne treatment, and appointments with a dermatologist). Photographs with examples of acne (eg, comedones, papules, pustules, and nodules) were provided to assist with self-recognition. In addition, the following validated HRQoL scales were administered: the dermatology life quality index (DLQI; for participants ≥ 16 years), children's DLQI (CDLQI; for participants < 16 years),^31^ and the Comprehensive Acne Quality of Life (CompAQ; all ages)^34^ referenced to the preceding week according to developer instructions. Linguistic translation and cultural adaptation were conducted in accordance with conventional methodology (TransPerfect, October 2019). Clinical experts (JT, BD) contributed to the development of the screening criteria, survey content, and selection of patient-reported outcome measures.

### Analysis

Descriptive statistics were used to summarize the survey (weighted) data set. For continuous variables, mean, standard error of the mean, and 95% confidence interval (CI) were calculated. For categorical variables, frequencies were reported. This study presents aggregate results for all study countries. DLQI, CDLQI, and CompAQ were scored according to their respective guidances. DLQI and CDLQI consisted of 10 questions with 4 possible answers for each scored from 0 to 3. The overall response scores were 0-30 with higher scores indicating greater impairment of HRQoL. The clinical interpretation of the DLQI scores was as follows: score 0-1 = no effect at all on the patient's life; 2-5 = small effect on the patient's life; 6-10 = moderate effect on the patient's life; 11-20 = very large effect on the patient's life; 21-30 = extremely large effect on the patient's life; a score >10 indicates that the patient's life is being severely affected by their skin disease.^31^ The clinical interpretation of the CDLQI scores is as follows: a 0-1 = no effect at all on the patient's life; 2-6 = small effect on the patient's life; 7-12 = moderate effect on the patient's life; 13-18 = very large effect on the patient's life; 19-30 = extremely large effect on the patient's life; a score >12 indicates that the patient's life is being severely affected by their skin disease.^35^ CompAQ consisted of 20 questions, each with 9 possible answers with a score range of 0-8. The total score range was 0-160 with higher scores indicating greater impairment in HRQoL.

Continuous variables were analyzed using Student *t* test or analysis of variance with 1 or more independent variables if 1 of the variables being compared had 2 or more levels (eg, age groups). Categorical variables were analyzed by chi-square independence test with Yates' correction and by Fisher's exact test. All tests were 2-tailed and *P* < .05 was considered statistically significant.

#### Multivariate regression

Regression models were used to evaluate the differences in the HRQoL between the respondents with F+T versus F. Variables identified in the literature as likely to be independently associated risk factors for acne severity and impact on HRQoL were included in the multivariate analysis (ie, age, sex, urban vs rural residence and country of residence, family history of acne, and acne severity grade at each body site). Country was modeled as the primary sampling unit to account for clustering of data at the country level. Odds ratios (ORs) with 95% CI were generated. The level of significance was set at *P* < .05. STATA version 15 (StataCorp LLC) was used for analyses.

## Results

### Demographic and clinical characteristics

A total of 1309 respondents consented to participate in the study and were allocated into 2 study groups: F+T (n = 694) and F (n = 615). Demographic and clinical characteristics of the participants are shown in Table I. There were no significant differences in terms of age, sex, or other demographic characteristics between the F and F+T groups (Table I), nor among the F+T group with acne on the face and chest alone versus acne on the face and back alone (data not shown). Demographic and clinical characteristics were similar across age groups (ie, <16 years vs ≥16 years), but there were more female adults in the older age group (36.8% vs 50.9%, respectively, *P* = .020), and the duration of facial and truncal acne was significantly longer in the older age group (+6.8 years [*P* = .001] and +6.5 years [*P* = .001] for facial and truncal acne, respectively) (data not shown).

**Table I:** Population demographics and acne characteristics

	F+T group	F group
	N = 694	N = 615
Age (years), mean (95% CI)	18.71 (17.3-20.1)	18.50 (17.8-19.2)
Age <16 years, n (%)	288 (46.8%)	333 (48.0%)
Sex, n (%)		
Males	385 (55.5%)	349 (56.8%)
Females	309 (44.5%)	266 (43.2%)
Type of residence, n (%)		
Urban	412 (59.4%)	364 (59.2%)
Suburban	201 (29.0%)	182 (29.7%)
Rural	80 (11.6%)	69 (11.1%)
Country, n (%)		
United States	323 (46.6%)	293 (47.6%)
Canada	33 (4.7%)	45 (7.2%)
Brazil	82 (11.9%)	86 (13.9%)
Germany	80 (11.6%)	60 (9.7%)
France	121 (17.4%)	79 (12.9%)
Italy	53 (7.7%)	53 (8.6%)
Clinical characteristics of acne		
Family history, n (%)		
Yes∗	581 (85.4%)	462 (78.0%)
Age at onset, mean (95% CI)		
Facial acne∗	12.6 (12.3-13.0)	13.1 (12.8-13.5)
Truncal acne	13.1 (12.7-13.5)	NA
Acne duration at time of survey completion (years), mean (95% CI)		
Facial acne	6.1 (4.5-7.7)	5.5 (4.4-6.5)
Truncal acne	5.6 (4.3-6.8)	NA
Current acne severity: Face, n	694	615
Almost clear	0	0
Mild	312 (45.0%)	310 (50.4%)
Moderate	249 (35.9%)	240 (39.1%)
Severe	113 (16.2%)	57 (9.3%)
Very severe	20 (2.9%)	8 (1.3%)
Current acne severity: Back, n	644	0
Almost clear	7 (1.0%)	
Mild	208 (32.0%)	
Moderate	287 (44.5%)	
Severe	119 (18.4%)	
Very severe	24 (3.7%)	
Current acne severity: Chest, n	317	0
Almost clear	46 (14.7%)	
Mild	123 (38.7%)	
Moderate	91 (28.8%)	
Severe	48 (15.3%)	
Very severe	8 (2.6%)	

*F*, Facial acne only group; *F+T*, facial and truncal acne group.

∗ *P* values for the comparison of F+T versus F groups significant at <.05.

F+T respondents reported acne involvement on the face (100%), chest (45.6%), and back (92.8%) at the time of questionnaire completion; 54.3% of F+T respondents had acne on the face and back only, 7.3% had acne on the face and chest only, and 38.5% had acne on all 3 sites (ie, face, chest, and back). Table II presents the proportion of respondents with acne on the face and back and/or chest by acne severity.

**Table II:** Correlation between the severity grade of acne on the face versus the back and chest

Severity of facial acne	Without acne on the back			Without acne on the chest			Acne on the chest and back		
	Severity of acne on the chest			Severity of acne on the back			Severity of acne on the chest and back		
	Mild	Moderate	Severe/very severe	Mild	Moderate	Severe/very severe	Mild	Moderate	Severe/very severe
Mild, n (%)	7 (1.0%)	4 (0.6%)	0 (0%)	112 (16.1%)	65 (9.4%)	7 (1.0%)	66 (9.5%)	2 (0.3%)	2 (0.3%)
Moderate, n (%)	5 (0.7%)	21 (3.0%)	1 (0.1%)	14 (2.0%)	96 (13.8%)	18 (2.6%)	7 (1.0%)	37 (5.3%)	6 (0.9%)
Severe/very severe, n (%)	2 (0.3%)	3 (0.4%)	6 (0.9%)	4 (0.6%)	17 (2.4%)	44 (6.3%)	3 (0.4%)	3 (0.4%)	35 (5.0%)

A higher proportion of F+T respondents self-reported severe or very severe facial acne compared with F respondents (19.2% vs 10.6%; *P* = .024) (Table I).

### Impact on HRQoL

The impact of acne on all HRQoL scales was significantly higher in the F+T respondents than in the F respondents (ie, mean CDLQI scores of 15.12 [95% CI 11.6-18.6] and 12.47 [95% CI 9.8-15.2], respectively [*P* = .001]; mean DLQI scores of 12.85 [95% CI 11.5-14.2] and 10.78 [95% CI 10.1-11.4], respectively [*P* = .011]; mean CompAQ scores of 101.4 [95% CI 89.7-113.0] and 87.3 [95% CI 79.6-94.9], respectively [*P* = .014]).

The prevalence of those reporting CDLQI scores indicative of “very large” or “extremely large” HRQoL impact (ie, total score > 12) was 61.3% versus 45.2% for F+T versus F (*P* = .001); the prevalence of those reporting DLQI scores of “very large” or “extremely large” HRQoL impact (ie, total score > 10) was 57.3% versus 44.5% for F+T versus F (*P* = .015). This difference remained significant in multivariate models in which the F+T respondents were almost twice as likely to have scores in the range of “very large” or “extremely large” impact on HRQoL compared with the F group (DLQI: OR F+T vs F = 1.61 [95% CI 1.02-2.54], *P* = .042; CDLQI: OR 1.86 [95% CI 1.10-3.14], *P* = .028) (Table III).

**Table III:** Odds ratios (ORs) for a score in the range of “very large” impact of facial and truncal acne in HRQoL (per CDLQI and DLQI) in adjusted logistic regression models with age, sex, acne location, and severity as explanatory variables

Explanatory variables	CDLQI score >12		DLQI score >10	
	Adjusted OR (95% CI)	*P* value	Adjusted OR (95% CI)	*P* value
Acne on both the face and trunk (F+T vs F)	1.86 (1.10 to 3.14)	.028	1.61 (1.02 to 2.54)	.042
Female vs male	1.07 (0.72 to 1.60)	.672	0.93 (0.49 to 1.73)	.766
Family history of acne: Yes	1.93 (0.94 to 3.97)	.067	1.21 (0.78 to 1.89)	.317
Urban vs rural residence	2.06 (0.96 to 4.42)	.058	1.84 (1.02 to 3.33)	.046
Unit increase in acne severity on face	2.31 (1.26 to 4.24)	.017	2.25 (1.93 to 2.64)	.001
Unit increase in acne severity on chest	1.99 (1.32 to 3.02)	.008	2.20 (1.14 to 4.22)	.027
Unit increase in acne severity on back	2.40 (1.15 to 5.00)	.028	2.11 (1.62 to 2.75)	.001

Country was also included in the adjusted analyses. Acne severities at each body site were not included together in the same model because of collinearity.

*CDLQI*, Children's dermatology life quality index; *CI*, confidence interval; *DLQI*, dermatology life quality index; *F*, facial acne only; *F+T*, combined facial and truncal acne; *OR*, odds ratio.

**Fig 1:**
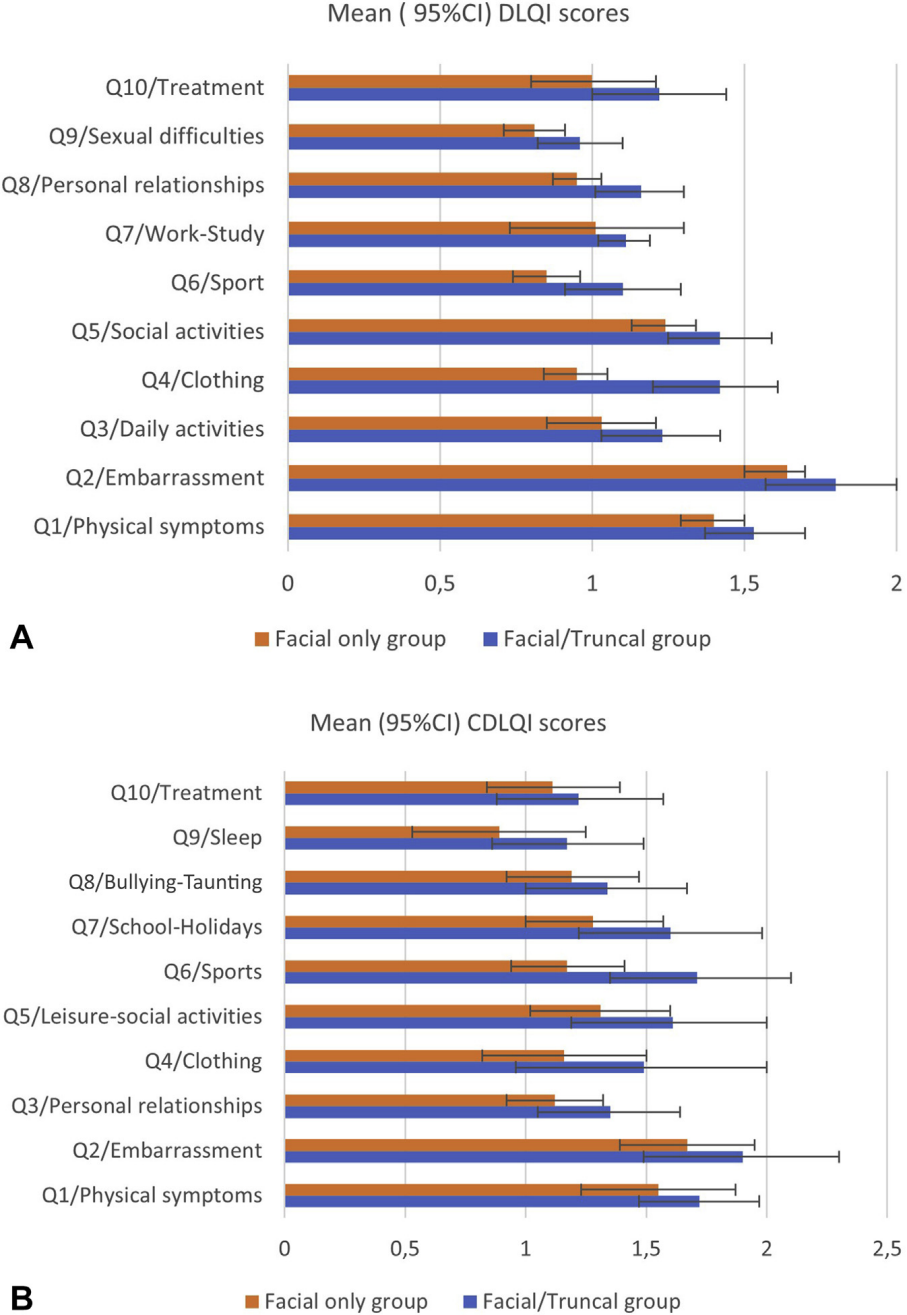
**A**, DLQI means (95% CI) for the “F+T” and “F” groups for individual questions. **B**, CDLQI means (95% CI) for the “F+T” and “F” groups for individual questions. *CDLQI*, Children's dermatology life quality index; *CI*, confidence interval; *DLQI*, dermatology life quality index; *F*, facial acne only; *F+T*, combined facial and truncal acne.

The majority of respondents (86.4% F+T and 91.5% F) reported being self-conscious because of their acne (*P* = .098 for the difference in proportions of F+T vs F). Significant differences between F+T and F were seen for both DLQI and CDLQI domains related to going out or clothing choice and participation in public activities and sports that revealed or made more visible their truncal acne (Fig 1, *A* and *B*).

### Other factors affecting acne-related impairment of HRQoL in facial and truncal acne

**Fig 2:**
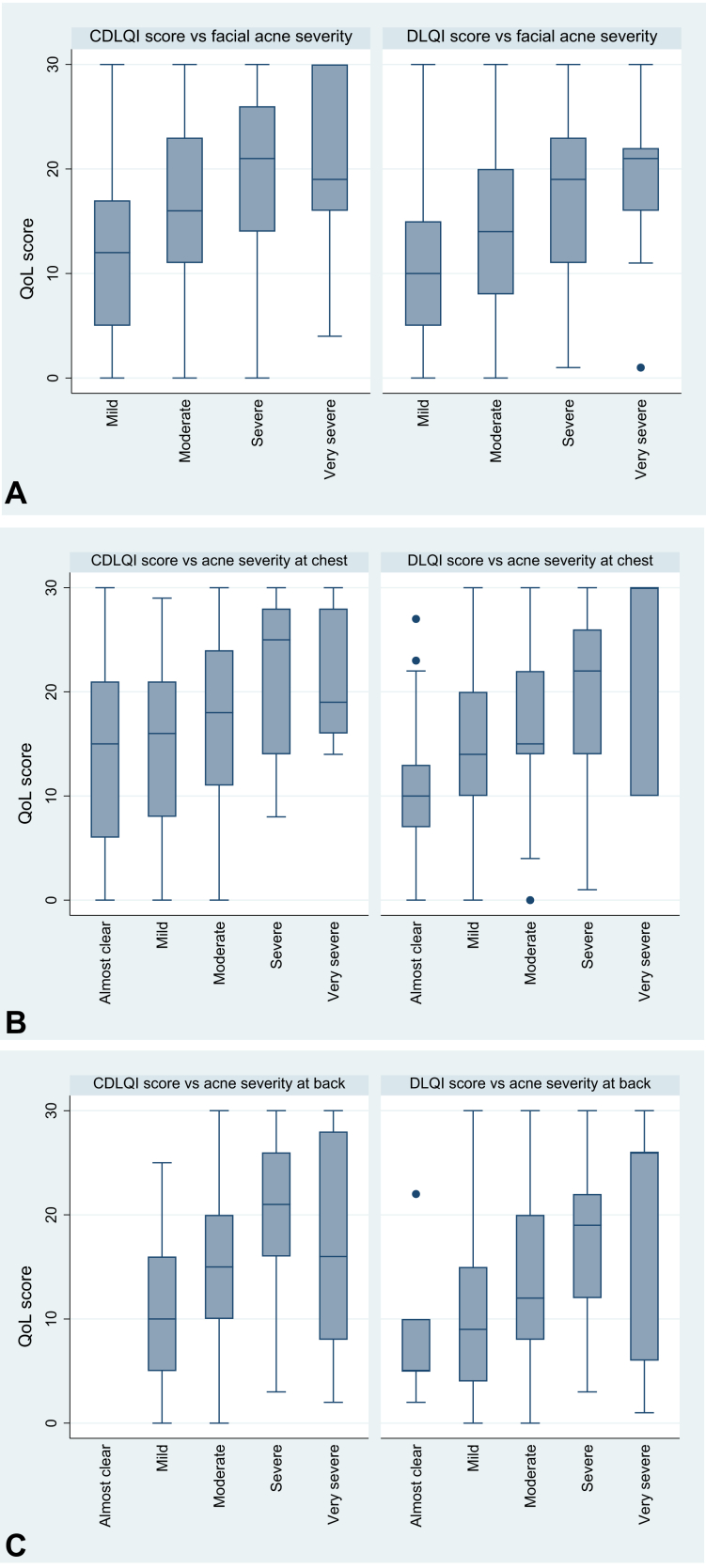
**A**, Distribution of total HRQoL scores (per DLQI and CDLQI) by facial acne self-rated IGA score. **B**, Distribution of total HRQoL scores (per DLQI and CDLQI) by chest acne self-rated IGA score. **C**, Distribution of total HRQoL scores (per DLQI and CDLQI) by back acne self-rated IGA score. *CDLQI*, Children's dermatology life quality index; *DLQI*, dermatology life quality index; *HRQoL*, health-related quality of life; *IGA*, Investigator Global Assessment; *QoL*, quality of life.

Irrespective of the acne location, the DLQI and CDLQI scores increased as the acne severity increased (Fig 2, *A* to *C*). This association remained significant in multivariate models after accounting for sex, country, type of residence, and family history of acne (Table III).

In stratified analysis, participants with mild-to-moderate facial acne who also had severe to very severe acne on the trunk reported significantly higher DLQI and CDLQI scores than respondents with mild-to-moderate acne on both the face and trunk (Table IV). This implied that, irrespective of the facial acne severity, severe acne of the back and/or chest was associated with additional HRQoL disability.

**Table IV:** Comparison of the DLQI and CDLQI individual item scores among participants with mild or moderate facial acne who suffered from either mild-moderate versus severe-very severe acne on their back and chest

	Mild-to-moderate facial acne							
	Mild/moderate vs severe/very severe acne on the back				Mild/moderate vs severe/very severe acne on the chest			
	Mean (95% CI)	Mean (95% CI)	Crude *P* value∗	Adjusted *P* value∗	Mean (95% CI)	Mean (95% CI)	Crude *P* value†	Adjusted *P* value†
DLQI	11.35 (9.6-13.1)	16.15 (13.7-18.6)	.005	.005	13.52 (11.4-15.6)	18.04 (4.0-32.1)	.291	.124
CDLQI	13.10 (9.7-16.5)	18.29 (13.1-23.5)	.004	.050	15.10 (11.8-18.4)	22.22 (11.3-33.1)	.012	.010

*CDLQI*, Children's dermatology life quality index; *CI*, confidence interval; *DLQI*, dermatology life quality index.

∗ *P* value for the comparison of the DLQI (or CDLQI) score between participants with mild-to-moderate acne on the back and severe/very severe acne on the back (irrespective of acne severity on the chest), keeping facial acne constant at mild-to-moderate acne.

† *P* value for the comparison of the DLQI (or CDLQI) score between participants with mild-to-moderate acne on the chest and severe/very severe acne on the back (irrespective of acne severity on the chest), keeping facial acne constant at mild-to-moderate acne.

## Discussion

In this study, combined facial and truncal acne was found to be associated with a greater impact on HRQoL than facial acne alone. The greater reduction in self-esteem observed with higher truncal acne severity, irrespective of the facial acne severity, implied that the visibility of facial acne is not the sole factor in acne-related psychosocial distress. These results are in line with studies showing that even if the impact of facial acne on attractiveness is thought to be a primary concern, the face and trunk each contribute to overall attractiveness in both sexes.^36^ In addition, satisfaction with the appearance of different body parts can impact both sexual experiences and satisfaction with those experiences.^37^^,^^38^ Prior HRQoL studies, which primarily focused on facial acne, may therefore inadequately represent the life experience of those who also have truncal involvement.

With increasing acne severity, DLQI scores for the self-perception, physical, social, and emotional domains also increased, indicating worse HRQoL. Those who perceived their acne as more severe were more self-conscious and had increased social avoidance behaviors. Nevertheless, even milder acne can be problematic, as almost half of the respondents reporting mild facial and truncal acne in this study also reported an adverse impact on HRQoL. These findings were consistent with previous studies.^29^^,^^39^^,^^40^ However, several studies have shown that clinician rating of disease severity does not always correlate with patient HRQoL.^41^, ^42^, ^43^ In this study, the self-rating of acne severity may have included aspects of objective disease severity and aspects of personal subjective experience, supporting the current view that a complete assessment of acne should not be limited to clinician-based measures but rather also include severity as perceived by the patient and patient-reported measures of HRQoL.^44^

Adolescents experience considerable psychological distress as a result of having acne, which may add to the emotional and psychological challenges experienced during this period.^40^^,^^45^ In this study, adolescents reported avoiding swimming and practicing other sports because of embarrassment, and schoolwork was negatively affected more often than in those in their late teens or young adulthood. Psychological issues such as dissatisfaction with appearance, embarrassment, self-consciousness, and lack of self-confidence that negatively influence the desire to participate in sports and schoolwork has been documented.^46^^,^^47^

We used HRQoL questionnaires adapted to each age group (ie, <16 and ≥16 years); therefore, we cannot compare results across age groups. Because the burden of acne may affect distinct age groups differently, this is an important consideration when attempting comparison with other studies that included a different range of age groups.

Our study had several limitations. We excluded respondents who did not have a prescribed acne treatment in order to ensure that respondents had a confirmed diagnosis of acne (by a health care professional). In addition, the severity of acne was self-rated by the participants. Nonetheless, provision of photographs representative of severity categories should have increased the objective accuracy of the reporting. The cross-sectional design of this study does not allow for temporal evaluation of acne impact. Time and cost requirements preclude such a longitudinal trial design.

The strengths of this study include the relatively large sample sizes of the F and F+T patient populations.

In conclusion, facial and truncal acne was associated with a greater impact on HRQoL than facial acne alone. HRQoL domains including emotional well-being, everyday life activities, participation in social activities and sports, and routine acne treatment were more affected in the F+T group than in the F group. Increasing severity of truncal acne increased the adverse impact on HRQoL irrespective of the severity of the facial acne. These results implied that, as for facial acne, early effective treatment of truncal acne is important to reduce disease-related psychosocial sequelae. Our findings should encourage the development of awareness programs and treatments to address truncal and facial acne.

## Conflicts of interest

Dr Tan has acted as a consultant for and/or received grants/honoraria from Bausch, 10.13039/501100009754Galderma, 10.13039/100004319Pfizer, Almirall, Boots/10.13039/100005153Walgreens, Botanix, Cipher, 10.13039/501100009754Galderma, Novan, 10.13039/100004336Novartis, Promius, 10.13039/501100004296Sun, Vichy. Dr Chavda is an employee of Galderma. Dr Beissert, Dr Cook-Bolden, Dr Harper, Dr Hebert, Dr Lain, Dr Layton, Dr Weiss, and Pr Dréno have acted as investigators and consultants for Galderma. Dr Rocha has acted as an advisor and/or speaker and received honoraria from Eucerin, Galderma, Johnson&Johnson and Leo Pharm.
